# Training a Model to Predict Asymbiotic Germination of Orchid Seeds on the Basis of Subfamily, Seed Morphology and Niche Profile

**DOI:** 10.3390/plants15172551

**Published:** 2026-08-22

**Authors:** Spyridon Oikonomidis, Anush Nersesyan, Hripsik Kosyan, Sonya Vardanyan, Costas A. Thanos

**Affiliations:** 1 Section of Botany, Department of Biology, National and Kapodistrian University of Athens, 15784 Athens, Greece; cthanos@biol.uoa.gr; 2 A. Takhtajan Institute of Botany, Acharyan 1, Yerevan 0063, Armenia; hr.kosyan@gmail.com (H.K.); sonyavardanyan2025@gmail.com (S.V.)

**Keywords:** E:S, germination, machine learning, orchids, seed morphology

## Abstract

Although asymbiotic orchid seed germination was first achieved in vitro in 1922, the prediction of germination requirements under in vitro conditions still remains complicated. To address this, we developed a machine learning framework to classify the ex situ asymbiotic germination potential of wild orchids into four discrete groups: Low (0–30%), Mid (31–50%), High (51–80%), and Max (81–100%). Models were trained on a dataset of 203 species, utilizing seed morphometrics—specifically, the embryo-to-testa (E:S) length ratio—alongside core ecological traits (subfamily, growth habit, habitat, and climate zone), as well as chemical scarification duration as a proxy of seed permeability. Validation leveraged novel germination and trait data from 26 taxa from Greece (17) and Armenia (9), published here for the first time. To mitigate class imbalance and prevent algorithmic bias toward highly germinating species, we applied inverse frequency weighting during training. Iterative testing of six algorithms revealed that the “Step 4” feature matrix (excluding climate zone and pretreatment duration) yielded the optimal predictive balance. K-Nearest Neighbor (KNN) and Support Vector Machine (SVM) emerged as the superior models, achieving overall accuracies of 44.4% and 61.1%, respectively, with both achieving 100% accuracy for low-germinating species. Finally, we synthesized a novel database compiling new seed morphometrics from Armenia (17 taxa), Greece (52 taxa), and the data from the literature (479 taxa). After filtering previously utilized species, we generated a prediction pool of 361 orchid taxa. Applying our Step 5 KNN and SVM models to forecast their germination behavior revealed distinct variations linked to ecological profiles. This high-accuracy framework, particularly for low-germinability groups, offers a powerful screening tool for ex situ conservation planning. The final trained models are compiled in the publicly available R (v. 4.6.0) package *OrchidGermClass*.

## 1. Introduction

The systematic study of seed germination dates back to the time of Theophrastus (371–287 B.C.), who is widely regarded as the father of botany [1,2]. Regarding orchids, their first mention in antiquity for medicinal purposes dates back to around 2700 B.C. in China [3]. However, their seeds were not formally described until after 1650 [4]. This was followed by the first documented experiments on symbiotic germination in 1909 [5] and the pioneering development of asymbiotic germination techniques in the laboratory about a century ago [6].

Orchidaceae is one of the largest plant families, comprising more than 28,000 species worldwide [7]. Because more than half of the orchids assessed to date fall into a threat category [8], the effective development of in situ and ex situ propagation practices is of paramount importance for their future conservation and survival.

The complexity of orchid germination requirements [9,10,11] stems largely from their dust-like seed size [12]. Orchid seeds are devoid of the endosperm nutrients necessary to support initial development prior to the photosynthetic stage [13]. Consequently, the development of predictive models for orchid seed germinability—utilizing readily available data—may play a pivotal role in the future of conservation planning.

Asymbiotic germination success within Orchidaceae is markedly inconsistent across different taxa. While certain species can germinate in carbon-deficient media—such as distilled water or water agar—development typically stalls at the rhizoid stage [13,14,15]. Most orchids depend on nutrient-dense artificial media for propagation, yet mature seeds from several terrestrial groups, notably *Cephalanthera* spp., remain unresponsive to current asymbiotic techniques [13,16,17]. These laboratory disparities likely reflect the ecological and symbiotic dependencies required for natural seedling recruitment in the wild [10,11,13].

Recent studies highlight the value of orchid seed morphometry not only for systematic purposes [18,19], but more importantly, for assessing germination behavior and classifying seed dormancy [11,20,21]. Formulating a predictive framework based on these observations will support future conservation efforts, allowing researchers to determine initial germination screening based on easily acquired morphometric measurements (such as the embryo-to-testa length ratio) alongside available distribution and life cycle data. The present study aims to develop such predictive models utilizing newly generated seed morphology and germination data from orchids of Greece and Armenia, supplemented by the available literature. By developing a high-accuracy germination classification model—with a primary focus on species exhibiting defiant germination (species that exhibit low germination percentages under asymbiotic culture but potentially high germination under symbiotic conditions) [11]—future studies will have a functional baseline. This framework will significantly reduce the time and effort required for ex situ experiments, which is especially critical given the prolonged germination durations characteristic of many orchid species [13].

## 2. Results

**Figure 1:**
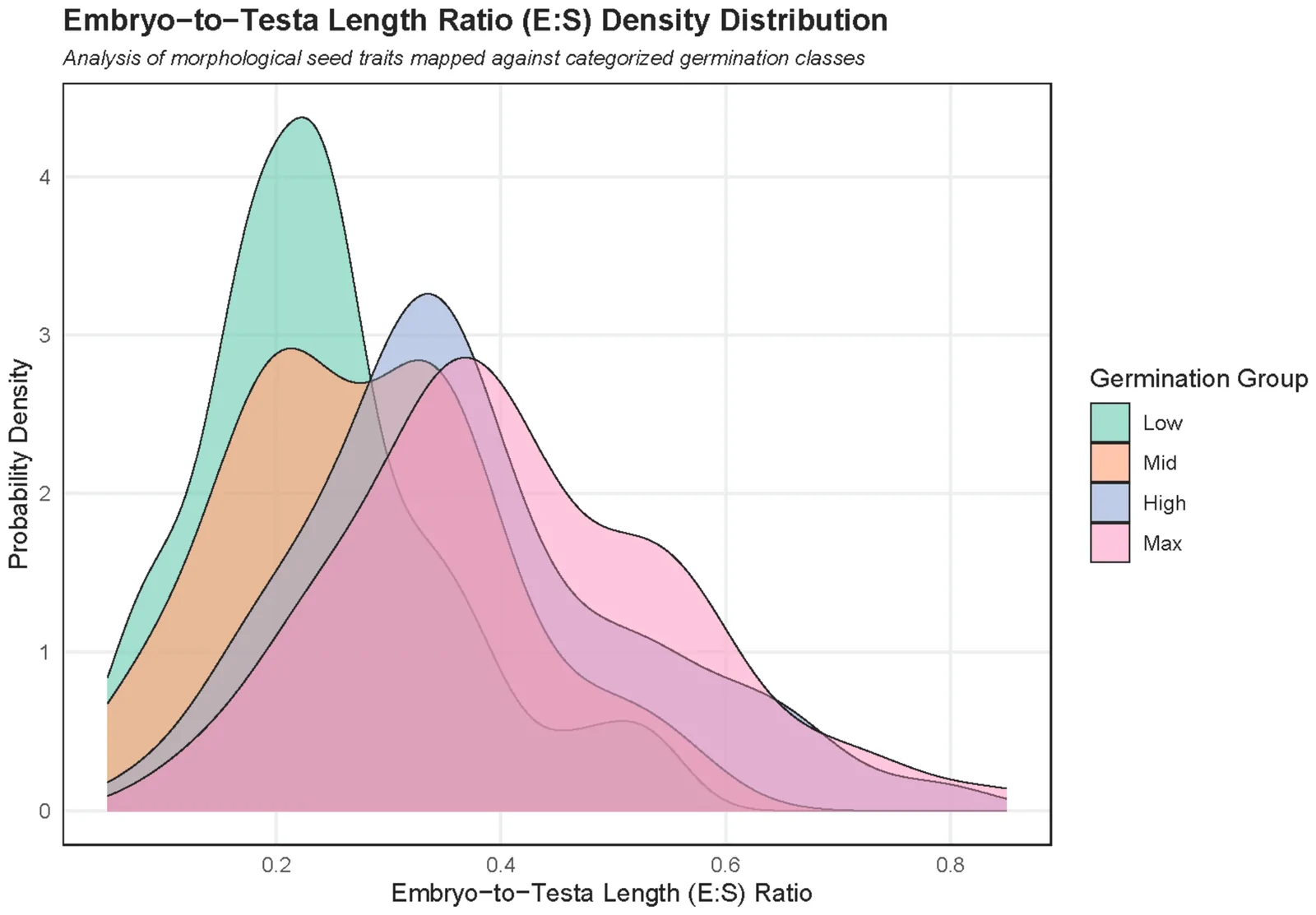
Embryo-to-testa length ratio (E:S) density distribution across the 4 germination classes for the testing dataset from [11].

The initial model training dataset comprised 203 unique orchid taxa. Categorization of final germination percentages (FG%) yielded an uneven distribution across the four defined groups: 69 taxa (34.0%) were classified as Max germinators (81–100%), 52 taxa (25.6%) as High germinators (51–80%), 18 taxa (8.9%) as Mid germinators (31–50%), and 64 taxa (31.5%) as Low germinators (0–30%). Morphometric evaluation of the training pool indicated an overall mean embryo-to-testa (E:S) length ratio of 0.33 ± 0.15 (range: 0.05–0.85). Segregation of this morphological parameter by germination performance demonstrated that taxa within the Max germination class possessed a mean E:S ratio of 0.41 ± 0.14, whereas taxa within the Low germination class exhibited a mean E:S ratio of 0.24 ± 0.10 (Figure 1).

**Figure 2:**
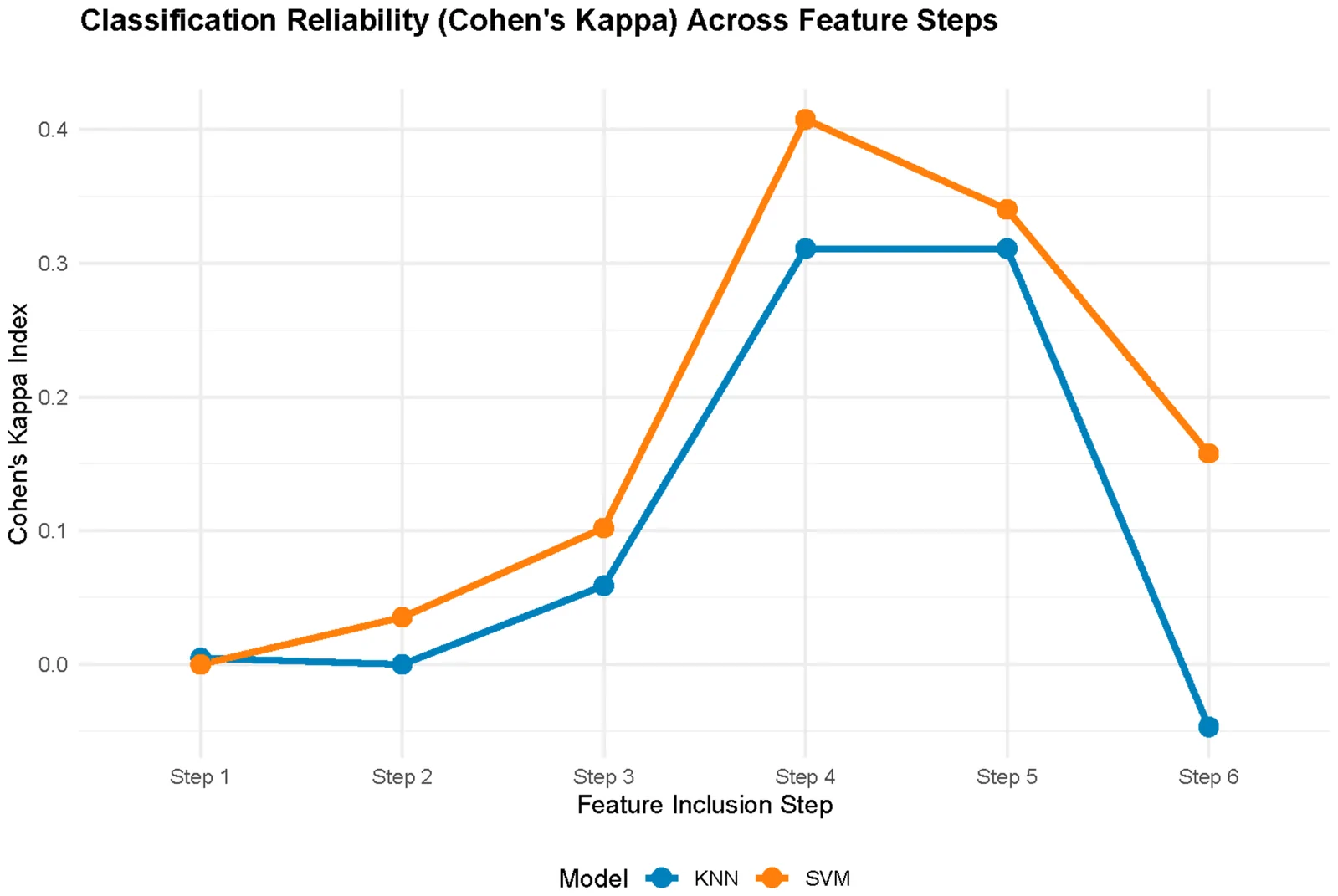
Cohen’s kappa across the six additive steps of model testing for the two best-performing models (KNN and SVM).

**Figure 3:**
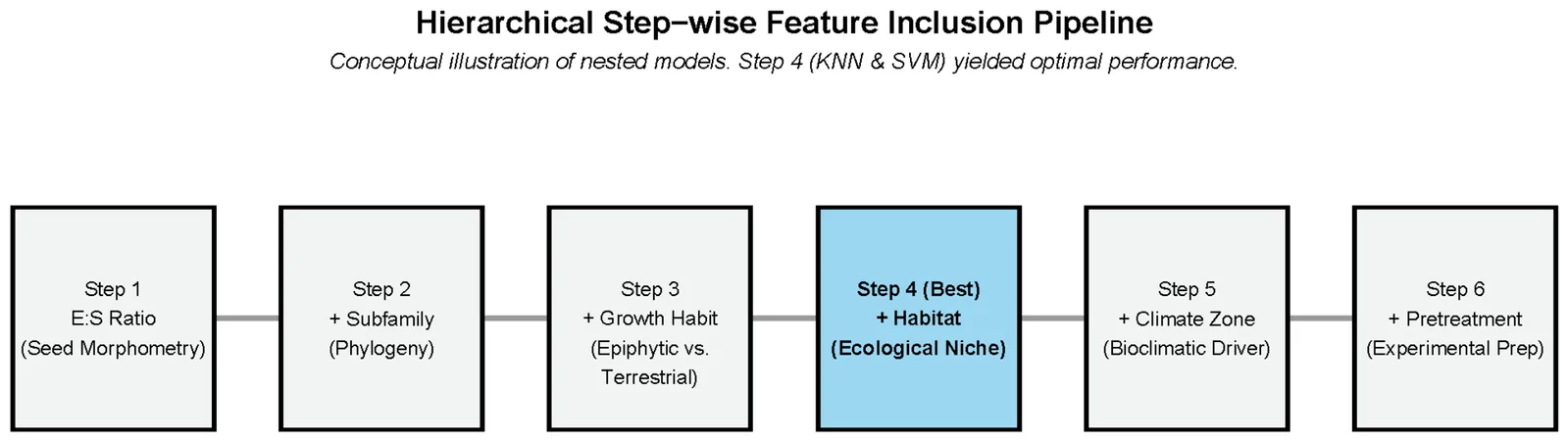
Hierarchical step-wise feature inclusion pipeline for the training and testing of the models.

**Figure 4:**
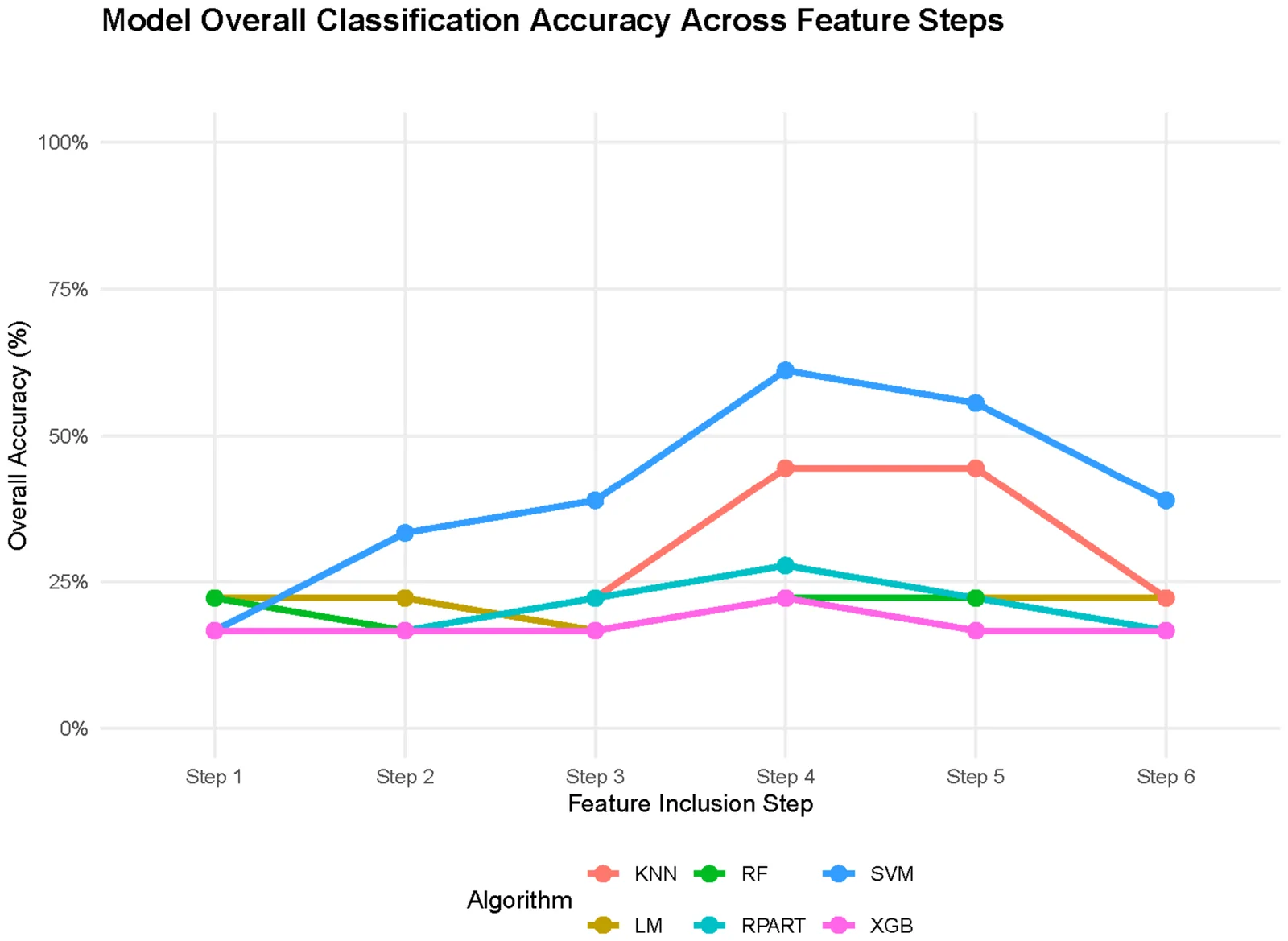
Overall percentages of stepwise accuracy of the tested models.

Evaluation of the six machine learning algorithms across the six hierarchical feature inclusion steps revealed a distinct peak in predictive performance at Step 4, utilizing a four-variable matrix (E:S ratio, subfamily, growth habit, and habitat). The baseline overall accuracy (Table 1) at Step 1, which relied solely on the E:S ratio, was 16.7% for SVM and 22.2% for KNN. Performance was maximized at Step 4, where the Support Vector Machine (SVM) algorithm achieved the highest overall metrics, with an accuracy of 61.1% and Cohen’s kappa of 0.4074 (Figure 2). The K-Nearest Neighbor (KNN) model at this step recorded an overall accuracy of 44.4% and a Kappa of 0.3110. Evaluation of class-specific sensitivity at the Step 4 threshold demonstrated that both the SVM and KNN algorithms correctly classified 100% of the taxa belonging to the targeted Low-germination group. Extending this evaluation to the full per-class breakdown and to all six algorithms (Table 2; Supplementary Table S5) confirmed SVM as the top-performing model across every summary metric (accuracy = 0.612, kappa = 0.407, macro-F1 = 0.442, and weighted-F1 = 0.579), followed by KNN (accuracy = 0.437, kappa = 0.311, macro-F1 = 0.327, and weighted-F1 = 0.351). Notably, none of the six models—including the four (LM, RPART, RF, and XGBoost; v3.2.1) trained with inverse-frequency class weighting—correctly classified any Mid-germination taxon in the independent test set (Mid F1 = 0 throughout). The weighted models also underperformed the unweighted SVM and KNN on every overall metric, indicating that at Step 4, algorithm choice was the dominant factor in classification performance rather than the weighting scheme. Critically, when evaluation was extended to account for the ordinal structure of the germination grades, model performance improved substantially. The quadratic-weighted Cohen’s kappa reached 0.858 for SVM and 0.790 for KNN at Step 4 (Table 2), compared with the unweighted values of 0.407 and 0.311 respectively. This large divergence between unweighted and quadratic-weighted Kappa indicates that when errors occur, they are predominantly adjacent-class misclassifications (e.g., High predicted as Max) rather than distant ones (e.g., Low predicted as Max), consistent with the regression-first architecture of the pipeline in which large cross-grade errors require correspondingly large continuous prediction errors on the FG% scale. RPART was the exception, showing minimal improvement under quadratic weighting (unweighted κ = 0.111; quadratic-weighted κ = 0.281), suggesting a higher proportion of non-adjacent misclassifications relative to the other algorithms. The subsequent inclusion of climate zone (Step 5) and pretreatment duration (Step 6) resulted in a decline in overall model reliability (for the stepwise feature inclusion see Figure 3), with Step 6 producing final overall accuracies of 38.9% for SVM and 22.2% for KNN (Figure 4).

**Figure 5:**
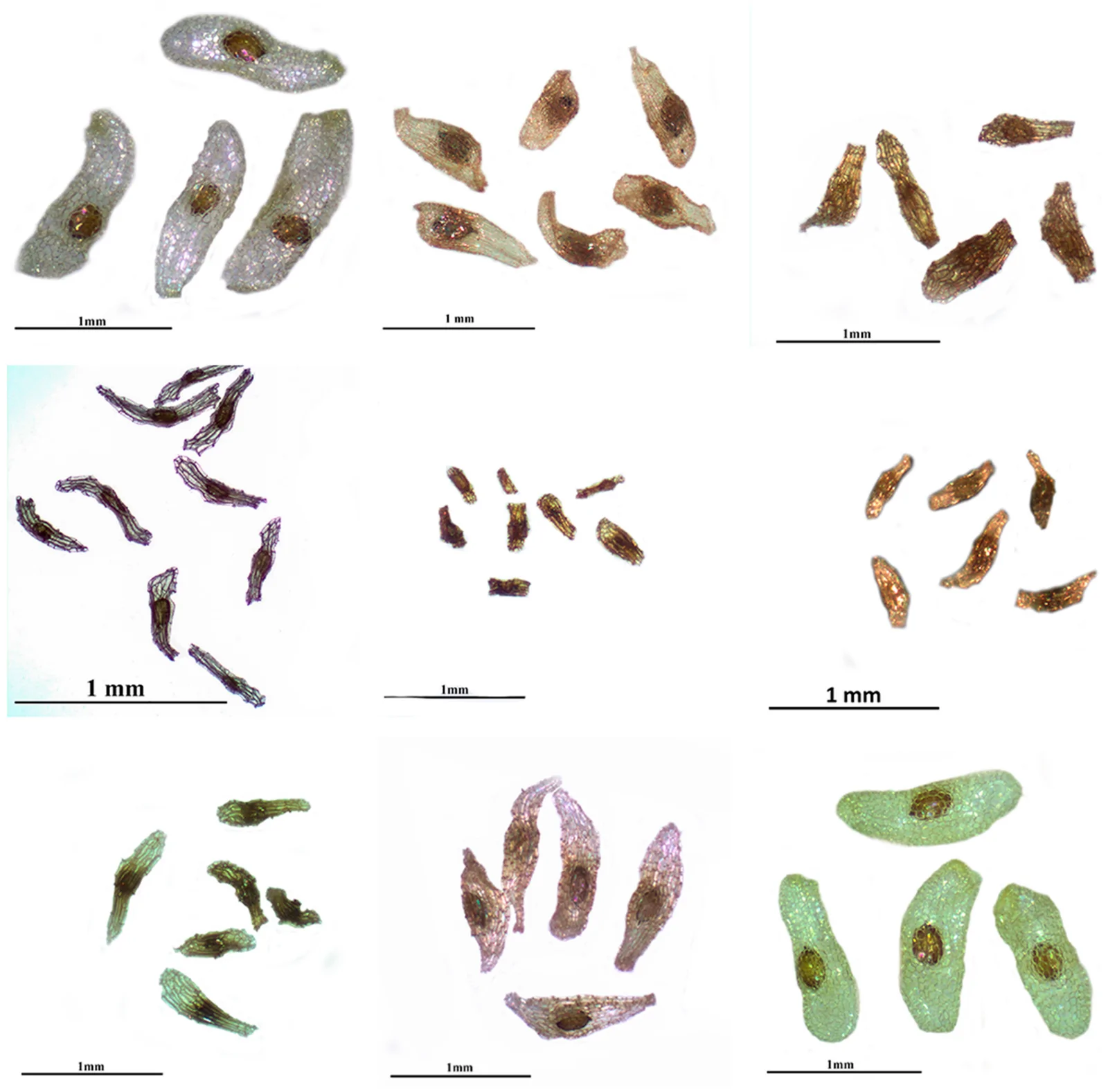
Seeds of selected orchid species used in the present analysis (from top and left): (1) *Cephalanthera damasonium*, (2) *Cephalanthera cucullata*, (3) *Dactylorhiza saccifera* subsp. *saccifera*, (4) *Neotinea maculata*, (5) *Orchis italica*, (6) *Spiranthes spiralis*, (7) *Ophrys umbilicata* subsp. *umbilicata*, (8) *Epipactis microphylla*, and (9) *Limodorum abortivum*.

Across all the taxa morphologically assessed from Armenia and Greece (Supplementary Table S2), the mean embryo-to-testa (E:S) length ratio exhibited a broad range, from a minimum of 0.196 ± 0.02 to a maximum of 0.653 ± 0.086. The lowest E:S ratios, indicating the most restricted embryo proportions relative to the overall seed length (Figure 5), were observed in *Cephalanthera damasonium* (0.196 ± 0.02, Armenia), *Cephalanthera longifolia* (0.198 ± 0.0316, Greece), and *Epipactis palustris* (0.198 ± 0.0461, Greece). Conversely, the highest E:S values within these novel records were recorded predominantly in members of the genus *Orchis*, specifically *Orchis quadripunctata* (0.653 ± 0.086, Greece), *Orchis anthropophora* (0.589 ± 0.09, Greece), and *Orchis provincialis* (0.518 ± 0.0803, Greece).

Asymbiotic germination trials were conducted on 26 taxa, encompassing 17 taxa from Greece and nine from Armenia. The final germination percentage (FG%) exhibited extreme variance across the tested taxa, ranging from complete failure (0%) to complete success (98.5%). Out of the 26 evaluated populations, 12 taxa (46.1%) fell into the critical Low-germination group (<30%). Within this Low group, eight taxa—*Cephalanthera damasonium*, *Epipactis helleborine* subsp. *helleborine*, *Epipactis microphylla*, *Limodorum abortivum*, *Neottia nidus-avis*, *Ophrys oestrifera* subsp. *oestrifera*, *Orchis mascula*, and *Platanthera chlorantha*—recorded an absolute germination failure of 0%. Conversely, the Max-germination group (>80%) comprised four taxa, with the highest overall performance observed in the Greek population of *Anacamptis morio* subsp. *caucasica*, which achieved a 98.5 ± 2.1% mean germination (Supplementary Table S3).

The finalized Step 4 KNN and SVM models were applied to an independent prediction pool comprising 361 untested orchid taxa (unique taxa filtered from the Supplementary Table S4 group). Taxonomic evaluation of this predictive dataset revealed a diverse composition encompassing 114 genera. The pool was dominated by members of the subfamily Epidendroideae (220 taxa; 60.9%), followed by the subfamilies Orchidoideae (111 taxa; 30.7%), Cypripedioideae (22 taxa; 6.4%), and Vanilloideae (8 taxa; 2.3%), while no representative from Apostasioideae was available for testing. The embryo-to-testa (E:S) length ratio spanned from a minimum of 0.018 (exhibited by *Dendrobium transparens*) to a maximum of 1.000 (exhibited by *Dendrobium macrostachyum* and *Polystachya concreta*), with an overall mean E:S ratio of 0.359 ± 0.203. Application of the Step 4 KNN and SVM models to the 361-taxon prediction pool yielded predictions across all four germination categories. KNN classified 42 taxa as Low, 41 as Mid, 219 as High, and 59 as Max; SVM classified 17 as Low, 23 as Mid, 278 as High, and 43 as Max. The breakdown of predictions by the categorical variables comprising the Step 4 feature set is summarized in Table 3. For SVM, all 17 Low-classified taxa belonged to Epidendroideae with a terrestrial/shaded profile, including *Gastrodia* spp. (*n* = 4), *Calanthe* spp. (*n* = 3), *Liparis* spp. (*n* = 3), *Cymbidium macrorhizon*, *Cymbidium nipponicum*, *Cephalanthera falcata*, *Corallorhiza mertensiana*, *Cremastra variabilis*, *Neottia japonica*, and *Oreorchis micrantha*. All 159 epiphytic taxa were classified by SVM as High (74.8%; *n* = 119) or Max (25.2%; *n* = 40), with no Low or Mid predictions. Orchidoideae (*n* = 111) were classified predominantly as High (97.3%), with no Low or Max predictions; Cypripedioideae (*n* = 22) as High (81.8%) or Mid (18.2%); and Vanilloideae (*n* = 8) exclusively as High (100%). All 43 Max-classified SVM taxa belonged to Epidendroideae, of which 40 (93.0%) were epiphytic. Open-habitat taxa (*n* = 176) received no Low predictions from SVM. For KNN, Low-classified taxa (*n* = 42) included representatives of Epidendroideae (*n* = 28), Cypripedioideae (*n* = 9), and Vanilloideae (*n* = 5), all with a terrestrial/shaded profile; all 22 Cypripedioideae were classified as Low or Mid by KNN, with no High or Max predictions, in contrast with the predominantly High classification by SVM. KNN similarly produced no Low predictions for epiphytic taxa, classifying them as High (65.4%; *n* = 104) or Max (33.3%; *n* = 53). All 59 KNN Max-classified taxa belonged to Epidendroideae, with 53 (89.8%) being epiphytic. Finally, based on the biological and ecological parameters of the models, the newly proposed R package *OrchidGermClass* rapidly classified the expected asymbiotic germination potential of unknown wild orchid species into one of four groups—Low (0–30%), Mid (31–50%), High (51–80%), or Max (81–100%)—while it also provided a final germination percentage prediction for each taxon of a provided data frame.

## 3. Discussion

Both Armenia and Greece host substantial plant biodiversity, serving as critical ecological hotspots for the Caucasus [22] and the Euro-Mediterranean regions, respectively [23]. Despite its relatively small geographic area, Armenia supports approximately 41 orchid taxa, establishing it as a prominent regional hotspot [22]. Similarly, the orchid flora of Greece comprises roughly 130 taxa—representing nearly half of all orchid taxa occurring in the Euro-Mediterranean region—ranking it as the second richest country in the area [23,24,25]. The immense, concentrated taxonomic diversity within these two regional floras as well as the global scale of the family Orchidaceae, combined with the escalating pressures of climate change and habitat fragmentation, underscores the urgent need for rapid, high-throughput conservation screening tools to safeguard the members of the family.

Asymbiotic orchid seed germination and propagation remain highly variable and idiosyncratic, governed by a complex array of customized growth media, chemical pretreatments, and incubation conditions [6,13]. Given the immense taxonomic breadth of the Orchidaceae, formulating a universal, empirically derived germination protocol is practically unfeasible on a purely experimental basis. While recent methodologies have attempted to streamline protocol formulation by systematically evaluating individual components [26], trial-and-error remains a severe bottleneck. This empirical limitation is particularly critical for conservation biology; with more than half of all evaluated orchid species currently categorized as threatened [8], the classical, purely experimental approach is too resource- and time-intensive to address the urgent demands of global ex situ conservation.

To overcome the empirical bottlenecks of traditional in vitro optimization, predictive modeling presents a transformative approach for conservation triage. Machine learning algorithms are particularly adept at resolving the complex, multidimensional relationships inherent to diverse biological datasets. By anchoring predictive frameworks on immutable morphological and ecological traits—specifically the embryo-to-testa (E:S) length ratio, phylogenetic lineage, and niche preferences —it becomes possible to forecast baseline physiological performance prior to any laboratory intervention. In the context of asymbiotic orchid propagation, deploying such algorithms to classify species into discrete germinability groups allows researchers to identify inherently hard-to-germinate taxa a priori. This predictive capability fundamentally shifts the ex situ conservation paradigm from reactive, resource-depleting experimentation toward proactive, targeted protocol design, ensuring that limited seed material and laboratory resources are allocated efficiently.

The germination behavior of orchids was previously placed along the mycoheterotrophy continuum [11,27] based on their seed morphology and ecologically relevant characteristics (e.g., habitat shadiness and biotic form). Building upon this foundation, the present work utilized existing datasets of orchid germination and seed morphological features [11], alongside newly generated data from orchids of Armenia and Greece, to develop a predictive germination classification tool. By integrating seed morphology, phylogeny, and the niche characteristics of the tested taxa, this framework forecasts the germination potential of newly evaluated species. To ensure this tool is highly accessible, we have made it publicly available through the *OrchidGermClass* R package. Ultimately, this package facilitates rapid species germination screening for untested taxa, significantly increasing experimental speed and reducing the potential effort and resource expenditure for researchers.

The hierarchical feature inclusion pipeline revealed that predictive performance peaked at Step 4 (utilizing E:S ratio, subfamily, growth habit, and habitat) before deteriorating in subsequent steps. Notably, the inclusion of chemical pretreatment duration (Step 6) resulted in a distinct decline in model accuracy and reliability (Cohen’s Kappa). We attribute this decline to reversed causal logic within the model architecture. Biologically, the requirement for chemical scarification is undeniably linked to intrinsic species traits [28,29], such as the degree of testa lignification, cuticular waxes, or the presence of a well-developed water-impermeable carapace (e.g., in *Cephalanthera* or *Gastrodia*). However, the specific duration of this pretreatment is an a posteriori experimental parameter, typically assigned empirically by researchers based on the known or suspected germination difficulty of the taxon. Consequently, forcing a human-assigned experimental proxy into a framework designed to predict that difficulty introduces logical circularity and statistical noise. As standardized methodologies [26] for quantifying inherent seed coat permeability become more prevalent (e.g., vital staining), this trait will have significant potential for future predictive refinement. Based on our model results, we introduce the *OrchidGermClass* R package, which deploys our two pre-trained models with the highest accuracy to forecast the germination potential of untested species. These models achieved an overall classification accuracy of 61% (SVM), driven substantially by consistently strong performance on the Low- and Max-germination groups, while demonstrating perfect precision and recall in identifying inherently low-germinating taxa. Performance on the Mid-germination group was poor across all evaluated algorithms, a limitation discussed further below. This flawless identification of low-germinating species is particularly powerful when contextualized with known biological barriers typical of their germination behavior, such as (1) strong mycoheterotrophic dependencies that necessitate symbiotic cultures or complex nutrient media with specific components, such as organic nitrogen [10,30]; (2) the presence of water-impermeable cover structures like carapaces [28,29]; and (3) notoriously prolonged incubation requirements [13]. Ultimately, *OrchidGermClass* provides an efficient a priori method to flag difficult taxa before initiating laboratory trials, offering researchers a data-driven basis for critical decision making regarding (1) nutrient media selection; (2) pretreatment scarification durations; and (3) the necessity of symbiotic versus asymbiotic germination strategies, where such options are available.

Regarding nutrient media selection, the comparative efficacy of organic versus inorganic nitrogen sources has historically been correlated with the specific ecological niches occupied by diverse orchid taxa [31,32]. Under this predictive framework, taxa classified in the low-germination group can be designated for symbiotic culture, where feasible, or sown on organic-rich nutrient media. This approach directly aims to satisfy their natural dependency on specific nutrient forms typically provided during fungal symbiosis [33].

Pretreatment duration significantly influences final germination performance; while adequate scarification increases coat (testa and carapace) permeability, excessive exposure can induce embryo toxicity and death. Historically, optimal pretreatment durations were determined through prolonged, trial-and-error germination experiments or extrapolated from established genus-level literature [13,34]. Recent methodological advances [24] utilize vital dye staining to streamline this decision-making process, identifying sensitive taxa where even minimal chemical exposure (<5 min) negatively impacts seed viability. In our predictive framework, such sensitive taxa—including *Neotinea maculata*, *Serapias lingua*, and several *Dactylorhiza* spp.—are consistently classified within the High- or Max-germination groups. These species are characterized by their requirement for minimal (<15 min) or even zero pretreatment to achieve successful asymbiotic germination [24]. In contrast, taxa requiring prolonged, aggressive scarification are almost exclusively partitioned into the Low-germination class. This low-germinating group prominently features species with highly developed, water-impermeable carapaces, such as *Cephalanthera falcata* and several *Gastrodia* spp. [28,29].

Beyond its immediate utility in germination forecasting, this study introduces the most comprehensive synthesized dataset of orchid seed morphological traits to date, encompassing 521 taxa (479 compiled from the literature and 42 novel records presented herein). While foundational for our predictive modeling, this dataset also offers substantial independent value for broader ecological inquiries, including seed dispersal dynamics and systematic relationships [19,35,36]. When our optimal predictive models were applied to this expanded species pool, the resulting classifications accurately mirrored established phylogenetic and ecological expectations. Fully or partially mycoheterotrophic genera that are notoriously hard to germinate asymbiotically—such as *Gastrodia*, *Tropidia*, *Cephalanthera*, *Neottia*, and *Nervilia* [11,13,27,37]—were distinctly partitioned into the Low-germination group. In contrast, taxa from genera widely recognized as highly germinable, including *Dactylorhiza*, *Dendrobium*, *Ophrys*, and *Cattleya* [13,20,26,38,39], were appropriately classified into higher success tiers. This qualitative genus-level consistency extends to ecological groupings across the full 361-taxon prediction pool. Both KNN and SVM produced no Low predictions for epiphytic taxa or open-habitat taxa, reflecting the ecological rarity of the germination barriers most strongly associated with the Low germination class—water-impermeable carapaces and obligate mycorrhizal dependency— among shade-adapted terrestrial lineages [11]. All SVM Low-classified taxa shared a terrestrial/shaded/Epidendroideae profile, and all Max-classified taxa across both models belonged exclusively to Epidendroideae, with the large majority being epiphytic (93.0% for SVM; 89.8% for KNN), consistent with the well-documented high asymbiotic germinability of several tropical epiphytic lineages in this subfamily [11]. The notable divergence between SVM and KNN predictions for Cypripedioideae—classified predominantly as High by SVM but exclusively as Low or Mid by KNN—and for Vanilloideae—classified entirely as High by SVM but as Low or Mid by KNN—reflects the limited representation of these subfamilies in the training set and is consistent with the expected extrapolation behavior of the two algorithms for feature space regions distant from dense training data, as discussed above in relation to cross-grade discrepancies. The convergence of both algorithms on biologically coherent prediction patterns across subfamily, growth habit, and habitat—groupings entirely independent of species or genus identity—provides systematic, quantitative support for the conclusion that the models are capturing generalizable trait–germination associations rather than taxonomic memory of training-set genera.

It should be noted that while the model returns both predicted germination percentages as well as germination classes for tested species, it is not capable of predicting germination rates for all orchids. While the model was trained on a relatively well-balanced dataset across all Orchidaceae [11], the resulting accuracy proves to be significantly higher for species that exhibit low germination percentages under asymbiotic conditions; thus, the model should be primarily used, in its current version, as a screening tool for identifying defiant germinating species and adjusting germination protocol development prior to seed sowing.

A methodological limitation of the present framework concerns the necessary dichotomization of ecologically continuous traits into binary categorical variables (growth habit: terrestrial/epiphytic; habitat: open/shaded). The habitat variable was not assigned arbitrarily: species were classified as open or shaded following the methodology of Oikonomidis and Thanos [11], which integrates qualitative habitat descriptions from the available literature with quantitative percentage shadiness estimates where corresponding distribution/location data existed, before collapsing this combined evidence into a binary designation. Nevertheless, we acknowledge that this final binarization does not preserve the full underlying gradient, and several taxa occupy intermediate or functionally distinct niches—including lithophytic and forest-edge habitats, as well as fully or partially mycoheterotrophic growth forms—that are not explicitly represented as separate categories. This simplification reflects a data sparsity constraint rather than a modeling preference: taxa exhibiting these intermediate or specialized states were represented by very few data points in our compiled dataset, and categories this rare are frequently absent altogether from one or more training folds during k-fold cross-validation, precluding any classifier from reliably learning their association with germination outcome. Notably, mycoheterotrophic dependency is not entirely obscured by this simplification: as discussed above, genera with strong mycoheterotrophic dependencies (e.g., *Gastrodia*, *Neottia*, and *Cephalanthera*) were, nonetheless, correctly and consistently partitioned into the Low-germination group, likely because their extremely low asymbiotic germination rates are also captured indirectly through other retained predictors (E:S ratio, subfamily, and phylogenetic placement), especially the E:S ratio, as described previously [11], which acts as a proxy of the placement of an orchid on the mycoheterotrophy continuum. Expanding these variables into multi-level or hierarchical categories—explicitly distinguishing lithophytic and fully mycoheterotrophic taxa and retaining percentage shadiness as a continuous predictor where location data permit—represents a clear priority for future model refinement as larger, more taxonomically balanced datasets for these underrepresented groups become available.

Three additional limitations of the validation design merit explicit acknowledgment. First, the number of germination replicates differs between the Greek (five Petri dishes per taxon) and Armenian (one Petri dish per taxon) test taxa, reflecting differences in available seed material for destructive experimentation. For the 17 Greek taxa, the standard errors of the mean germination percentage were generally low (range: 0.00–13.72%; mean: 3.51%), indicating that FG% estimates are reasonably stable across replicates for most taxa. However, three Greek taxa showed inter-replicate standard errors exceeding 11% (*Neotinea lactea*, SE = 13.72%; *Neotinea tridentata*, SE = 12.67%; and *Orchis militaris*, SE = 11.09%), all of which fall near class thresholds, meaning their germination class assignment is sensitive to replicate-level variability. For the nine Armenian taxa, the use of a single Petri dish precluded any assessment of within-taxon measurement precision, representing a genuine uncertainty in their FG% estimates that cannot be quantified from the available data. Future validation efforts should prioritize multi-replicate germination trials for all taxa, particularly those whose mean FG% falls near class boundaries. Second, the class distribution of the 26-taxon validation set—Low: 46.2% (*n* = 12), Mid: 23.1% (*n* = 6), High: 15.4% (*n* = 4), and Max: 15.4% (*n* = 4)—reflects the biological reality of orchid germination distributions in the European and Armenian Orchidoideae-dominated floras, where low asymbiotic germination is genuinely a common outcome, rather than a deliberate sampling decision where collection viability immediately after collection can frequently be low [40]. Nevertheless, the small absolute sample sizes for the High and Max classes limit statistical power for evaluating model performance in those categories, consistent with the performance patterns observed across all six algorithms (Table 2; Supplementary Table S5). Third, all 12 genera represented in the validation set are also present in the training set. While the predictive models do not use genus or species identity as input variables—the only phylogenetic signal encoded is subfamily, a coarse five-level categorical variable grouping hundreds of genera—the possibility that subfamily-level patterns in E:S ratio and ecological traits captured during training partially explain validation performance cannot be fully excluded. The 361-taxon case study includes genera entirely absent from the training set, such as *Lecanorchis*, providing a partial test of out-of-genus generalization, though without ground-truth germination data for verification. Validation with taxa from genera and subfamilies not represented in the training set, using new germination data, remains a clear priority for model refinement.

In conclusion, the present study provides a predictive model highly accurate for low-germinating species, publicly available via the *OrchidGermClass* R package, for classifying orchid germination potential based on readily available ecological niche and systematics data, alongside easily acquired seed morphological measurements. As described above, this model facilitates a rapid initial screening of germination requirements for untested orchid taxa. This significantly boosts the conservation potential for this threatened family by reducing the time and cost associated with blind experimental testing. Nevertheless, future improvements to these classification techniques are necessary, particularly to facilitate the development of species-specific protocol predictions. To achieve this, a more standardized methodological approach to orchid germination must be adopted to ensure consistency in predictive factors, such as pretreatment duration, germination conditions (e.g., growth medium and incubation period), and the developmental criterion used to define successful germination (e.g., rhizoid emergence vs. protocorm formation). Because our models predict the continuous final germination percentage before applying class thresholds, such criterion-driven noise enters the training data directly; the resulting classification is buffered against this noise only to the extent that a given error does not cross a class boundary, which likely contributes to the comparatively lower classification accuracy observed for the intermediate Mid and High groups relative to the Low group. This disparity was most pronounced for the Mid class specifically: across all six evaluated algorithms, not a single Mid-germination taxon was correctly classified in the independent test set (Table 2), a limitation we attribute primarily to the small absolute number of Mid-class taxa available (18 of 203 of the training dataset) rather than to algorithm choice, since inverse-frequency class weighting—applied specifically to counteract this imbalance—did not improve Mid-class detection in any weighted model. This limitation extends to the 361-taxon case study: although neither Step 4 model correctly identified a Mid-germination taxon during evaluation, both nonetheless assigned a proportion of the prediction pool to the Mid category. Given that only 2 Mid-class taxa were available for evaluation, this is not necessarily contradictory—too few Mid taxa were tested to establish confidently whether either model can recognize the class at all—but as the case study applied the trained models to taxa lacking empirical germination data, these Mid assignments cannot be verified against the resulting model performance. Predictions falling in the Mid category, including those generated for the 361-taxon pool, should, therefore, be treated with greater caution than Low-category predictions.

Discrepancies between KNN and SVM predictions for individual taxa in the case-study pool—including instances where the two algorithms differ by two germination grades, as observed for several (5/7) *Lecanorchis* species—warrant explicit interpretation. Across the full 361-taxon prediction pool, KNN and SVM predictions were in perfect agreement for 276 taxa (76.5%), differed by one grade for 72 taxa (19.9%), and differed by two grades for only 13 taxa (3.6%), indicating that substantial cross-grade disagreement is the exception rather than the rule. Where discrepancies do occur, they arise from the fundamentally different geometric decision boundaries of the two algorithms: KNN assigns predictions based on the local neighborhood of the most similar training taxa, whereas SVM defines a global hyperplane maximizing the margin between classes in the transformed feature space. For taxa whose ecological profile places them in a region of feature space sparsely or entirely unrepresented in the training data—as is the case for *Lecanorchis*, a genus with no representatives in our training set—the two algorithms extrapolate differently, producing divergent continuous FG% predictions that, after class-threshold assignment, fall into non-adjacent grades. Large cross-grade discrepancies should, therefore, be interpreted as a signal of low model confidence for that specific taxon rather than as a systematic inconsistency of the framework. Users are encouraged to consult the underlying continuous raw FG% predictions alongside the assigned class labels, particularly for genera absent from the training set or whose predicted values fall near a class boundary, and to supplement model output with expert ecological assessment in such cases.

**Figure 6:**
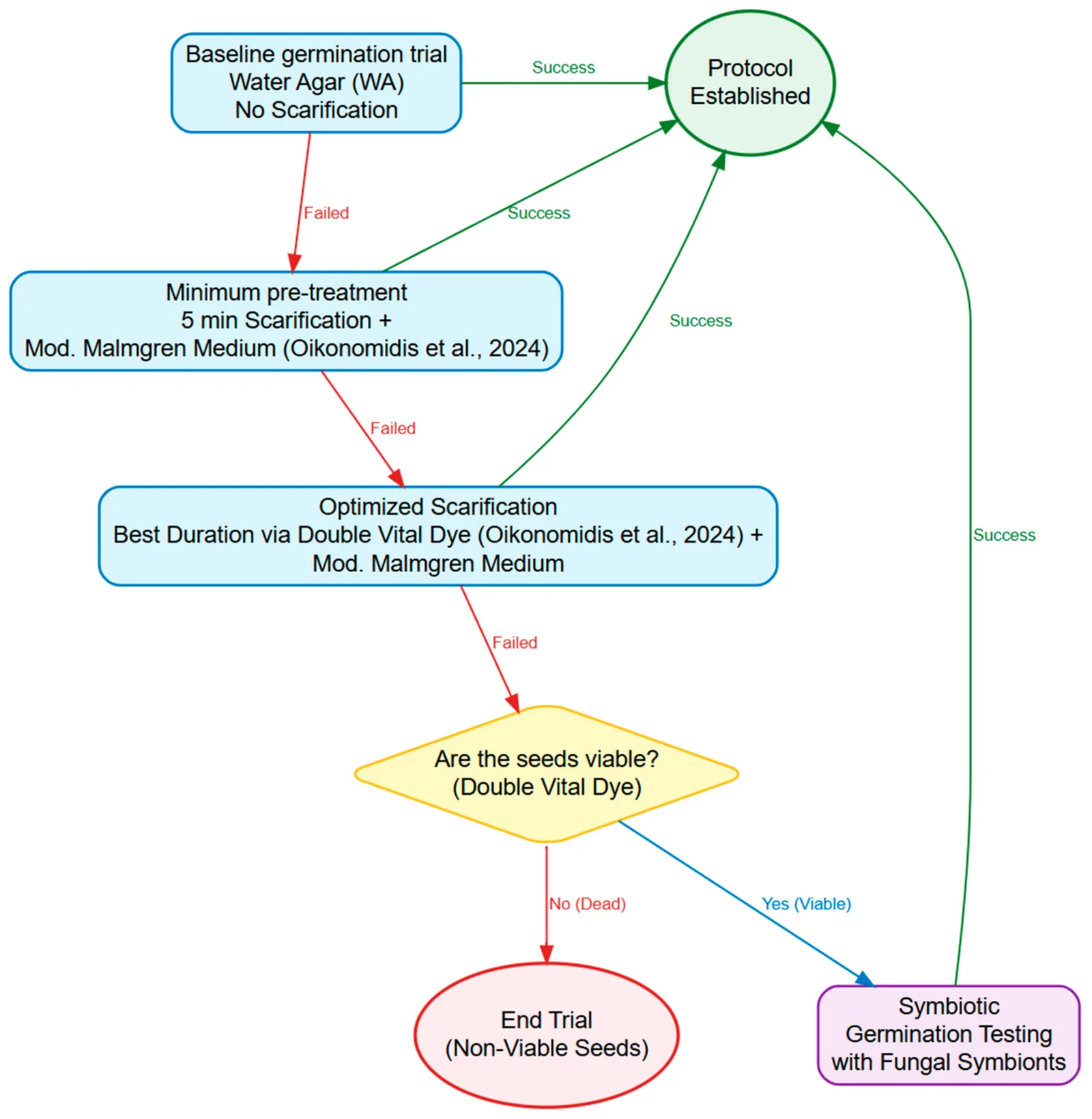
Orchid germination workflow for future development of a germination protocol prediction for orchid taxa [26].

The great variety of media used in orchid germination can significantly affect the accuracy of the developed models; for example, in our present study, our testing dataset (new data from our research teams) used the same germination medium, but the literature data used, in most cases, a different medium for each species. Consequently, we propose a tiered germination screening framework (Figure 6) designed to systematically evaluate the following: (1) intrinsic germinability with and without nutrient media (Rasmussen, 1995) [13] (as a basal medium, the modified Malmgren used in the current study provided a good initial germination medium for several orchid species); (2) the necessity and duration of chemical pretreatments [26]; and (3) fungal symbiotic dependencies. Across all such experimental trials, assessing baseline seed viability prior to testing remains mandatory, given the frequently low viability observed in many orchid taxa even immediately following collection from ostensibly sound seeds [40]. Crucially, advancing these predictive tools necessitates a paradigm shift in botanical publishing. We must highlight the urgent need to publish negative germination results. While data from failed germination tests are frequently hindered by publication bias in peer-reviewed journals, they remain an invaluable, highly underutilized resource for training robust machine learning frameworks.

## 4. Materials and Methods

The dataset utilized for model training and validation was synthesized from multiple sources to encompass a broad phylogenetic and ecological range within the Orchidaceae family. The primary training set comprised 203 orchid taxa with known germination and morphometric data, primarily sourced from a previous work [11]. This was supplemented by a dataset consisting of 69 taxa (62 unique and 7 from both countries) from Armenia (17 taxa) and Greece (52 taxa), for which seed morphology traits are reported for the first time in this study and represent approximately 1/3 of the orchid flora of each country. From these taxa, 26 were selected and also tested for their germination behavior (9 from Armenia and 17 from Greece); similarly, the germination protocols and results for these taxa are reported here. These two groups of 69 taxa (for morphological measurements) and 26 taxa (both germination and morphological measurements) were used for the testing of the model. Finally, a collection of seed morphology data was compiled from the literature for 479 taxa for further use as a case study for the model’s predictive capabilities (Supplementary Table S1).

For the germination of the taxa from Greece, we used 5 Petri dish replicates of approximately 100 seeds per Petri dish, while for Armenia, due to low seed availability for destructive experimentation, only 1 Petri dish was used. The seeds were first pre-treated in a 5% Ca(OCl)_2_ solution for 6–720 min depending on the taxon; the pretreatment duration was selected as suggested in a previous work [26] or based on the suggested treatment duration for closely related taxa [13]. After the pretreatment, the seeds were rinsed with distilled water under sterile conditions before sowing. For the asymbiotic germination tests, the modified basal nutrient medium Malmgren [26] was used, and the Petri dishes were stored in darkness at 20 °C and were monitored until no germination was observed for more than two weeks. In the present study, a seed was defined as germinated if rhizoids had begun to emerge from the embryonic body [41].

Morphometric data were obtained via light stereomicroscopic analysis of 30 mature seeds for each taxon and collection. For the new records from Greece and Armenia, seeds were photographed using a digital camera coupled with a stereomicroscope. Measurements included testa length (TL, mm), testa width (TW, mm), embryo length (EL, mm), and embryo width (EW, mm). The embryo-to-testa length (E:S) ratio was calculated as EL/TL for each individual seed to quantify the relative volume occupancy of the embryo within the seed coat.

For the machine learning model training, five categorical variables were assigned to each species in the dataset. (1) Subfamily: categorized as Apostasioideae (A), Cypripedioideae (C), Epidendroideae (E), Orchidoideae (O), or Vanilloideae (V). (2) Growth habit: classified as terrestrial (T) or epiphytic (E). (3) Habitat: classified as open (O) or shaded (S). (4) Climate zone: categorized as temperate (Te) or tropical (Tr). (5) Pretreatment duration: recorded as the duration (minutes) of chemical or physical scarification/bleaching applied prior to sowing. The variables were chosen based on the variables explored in a previous work [11]. The same variables/classification scheme was also used for the 479 literature records for further model testing. The designation of each species in each dichotomous variable followed the methodology suggested by Oikonomidis and Thanos [11], drawing both from species/taxa descriptions throughout the available literature as well as existing distribution data.

The target variable for all models was the final germination percentage (FG%). Given the disparities in FG reported throughout the orchid literature, germination data were also categorized into four discrete ordinal classes: (1) Low: 0–30%, (2) Mid: 31–50%, (3) High: 51–80%, or (4) Max: 81–100%. The class ranges were adapted to more practical experimental thresholds, based on the previously identified multimodal distribution of the training dataset [11].

All numerical features (E:S ratio and pretreatment duration) were centered and scaled (Z-score normalization) to ensure that feature magnitude did not bias the distance-based or gradient-based algorithms. Categorical variables were transformed into dummy variables for compatibility with numerical solvers.

Six algorithms were evaluated: Linear Regression (LM), K-Nearest Neighbor (KNN), Decision Tree (RPART), Support Vector Machine with a Radial Basis Function (SVM), Random Forest (RF), and eXtreme Gradient Boosting (XGBoost, v3.2.1). These six algorithms were selected to systematically benchmark parametric linear baselines (LM) against increasingly complex non-linear methods, capturing geometric proximity (KNN), boundary projection (SVM), hierarchical partitioning (RPART), and advanced ensemble aggregation (RF; XGBoost) to maximize predictive capacity. To determine the optimal feature set, an iterative six-step forward inclusion process was utilized. (1) Step 1: ES_ratio only, (2) Step 2: Step 1 + Subfamily, (3) Step 3: Step 2 + Growth Habit, (4) Step 4: Step 3 + Habitat, (5) Step 5: Step 4 + Climate Zone, and (6) Step 6: Step 5 + Pretreatment Duration. Although originally, all possible variable addition combinations were tested in the present work, we report the one resulting in the models with the highest achieved accuracy.

A significant challenge in orchid germination datasets is class imbalance, as the majority of experimental records represent species with high germination rates, while low-germinating species are underrepresented due to the seldom reporting of negative results in the literature. To prevent algorithmic bias toward the majority “Max” class, we implemented an inverse frequency weighting strategy during model training (see Supplementary Table S6 for the unweighted vs. weighted model version summarized results). Weights (Wi) were calculated for each class (c) according to the following formula:

$$w_{C}=\frac{N}{k\times n_{C}}$$

where N is the total number of samples, k is the number of classes, and n_c_ is the number of samples in class c. This inverse frequency weighting was applied to the training dataset for compatible tree-based and regression algorithms (Linear Regression, Decision Tree, Random Forest, and XGBoost). However, due to architectural constraints within the caret framework, K-Nearest Neighbor (KNN) and Support Vector Machine (SVM) were trained without explicit class weights, relying instead on centering, scaling, and their native geometric multi-dimensional separation properties. The inverse weighting improved, in several cases, the performance of the 4 models applied to (Supplementary Table S6) and, thus, only these versions are reported in the present study.

Cross-validation (5-fold) was employed during training to tune hyperparameters (k in KNN, cp in RPART, and tree depth in XGBoost). For the full list of hyperparameters, see Supplementary Table S7.

Model performance was assessed using overall accuracy (OA) and class-specific accuracy for the “Low” category. After establishing Step 4 as the most robust ecological model, it was deployed to forecast the germination behavior of a novel species pool.

To more comprehensively characterize model performance given the pronounced class imbalance in the training data (Mid = 8.9%), we additionally computed, for all six algorithms in the Step 4 feature set, the per-class precision, recall, and F1-score, together with macro-F1 and weighted-F1 (weighted by class prevalence) as overall summary statistics that explicitly account for imbalance. For classes with no correctly identified positive predictions, F1 was treated as 0 in the macro-F1 and weighted-F1 calculations rather than excluded, so that complete class failure was reflected in the summary statistics rather than masked by it.

To explicitly account for the ordinal structure of the germination classes, the quadratic-weighted Cohen’s Kappa was additionally computed for all six algorithms in Step 4, penalizing disagreements proportionally to the squared distance between predicted and actual class rank (Low = 1, Mid = 2, High = 3, and Max = 4), such that a Low→Max misclassification incurred a four-fold greater penalty than a Low→Mid misclassification.

To ensure the integrity of the predictive test, any taxa present in the original training or testing sets were filtered out. The final pool of 361 orchid taxa was processed to calculate mean E:S ratios. The Step 4 KNN and SVM models were utilized to generate germination class forecasts for these 361 taxa. All data processing, modeling, and visualization were performed in R (v.4.3.1) [42].

To facilitate the practical application of our predictive framework for ex situ conservation planning, the final, best-performing models were compiled into a publicly available R (v4.6.0) package, *OrchidGermClass* (V1.0, created 27 May 2026, accessible via the following GitHub repository at https://github.com/soikonomidis/OrchidGermClass (accessed on 27 May 2026)). The package integrates the optimized K-Nearest Neighbor (KNN) and Support Vector Machine (SVM) algorithms built on the optimal “Step 4” feature matrix. Utilizing centered and scaled predictors and relying on native geometric multi-dimensional separation, the package requires four easily obtainable inputs to execute predictions: the seed embryo-to-testa (E:S) length ratio, subfamily, growth habit, and habitat.

## Figures and Tables

**Table 1:** Overall percentage accuracy of each model and stepwise factor incorporation of the models.

Step	LM	KNN	RPART	SVM	RF	XGB
Step 1	22.2	22.2	16.7	16.7	22.2	16.7
Step 2	22.2	16.7	16.7	33.3	16.7	16.7
Step 3	16.7	22.2	22.2	38.9	16.7	16.7
Step 4	22.2	44.4	27.8	61.1	22.2	22.2
Step 5	22.2	44.4	22.2	55.6	22.2	16.7
Step 6	22.2	22.2	16.7	38.9	16.7	16.7

**Table 2:** Overall and per-class classification performance of six machine learning algorithms (LM, KNN, RPART, SVM, RF, and XGBoost) in the Step 4 feature set.

Model	Accuracy	Kappa	Quad-W Kappa	Macro_F1	Weighted_F1	Low_F1	Mid_F1	High_F1	Max_F1
Step4_LM	0.125	0	0.547	0.0769	0.0385	0	0	0.3077	0
Step4_KNN	0.437	0.311	0.790	0.3269	0.351	1	0	0.3077	0
Step4_RPART	0.250	0.111	0.281	0.2083	0.2448	0.3333	0	0.25	0.25
Step4_SVM	0.612	0.407	0.858	0.441	0.578	1	0	0.222	0.545
Step4_RF	0.187	0.058	0.609	0.160	0.142	0.333	0	0.307	0
Step4_XGB	0.125	0	0.547	0.076	0.038	0	0	0.307	0

**Table 3:** Breakdown of Step 4 SVM and KNN germination class predictions for the 361-taxon prediction pool by subfamily, growth habit, and habitat. Values are taxon counts with row percentages in parentheses.

Variable	Group	*n*	SVM Low	SVM Mid	SVM High	SVM Max	KNN Low	KNN Mid	KNN High	KNN Max
Subfamily	Epidendroideae	220	17 (7.7%)	16 (7.3%)	144 (65.5%)	43 (19.5%)	28 (12.7%)	11 (5.0%)	122 (55.5%)	59 (26.8%)
	Orchidoideae	111	0 (0%)	3 (2.7%)	108 (97.3%)	0 (0%)	0 (0%)	14 (12.6%)	97 (87.4%)	0 (0%)
	Cypripedioideae	22	0 (0%)	4 (18.2%)	18 (81.8%)	0 (0%)	9 (40.9%)	13 (59.1%)	0 (0%)	0 (0%)
	Vanilloideae	8	0 (0%)	0 (0%)	8 (100%)	0 (0%)	5 (62.5%)	3 (37.5%)	0 (0%)	0 (0%)
Growth habit	Terrestrial	202	17 (8.4%)	23 (11.4%)	159 (78.7%)	3 (1.5%)	42 (20.8%)	39 (19.3%)	115 (56.9%)	6 (3.0%)
	Epiphytic	159	0 (0%)	0 (0%)	119 (74.8%)	40 (25.2%)	0 (0%)	2 (1.3%)	104 (65.4%)	53 (33.3%)
Habitat	Open	176	0 (0%)	2 (1.1%)	158 (89.8%)	16 (9.1%)	0 (0%)	17 (9.7%)	141 (80.1%)	18 (10.2%)
	Shaded	185	17 (9.2%)	21 (11.4%)	120 (64.9%)	27 (14.6%)	42 (22.7%)	24 (13.0%)	78 (42.2%)	41 (22.2%)

## Data Availability

The data files and code necessary for replicating the current analysis are included in the present study or are submitted as Supplementary Materials in the following repository: https://github.com/soikonomidis/Orchid_predict_manuscript_supplementary_material_2026 (accessed on 27 May 2026). The raw (non-summarized) datasets are available from the authors upon reasonable request.

## Supplementary Materials

The following supporting information can be downloaded at https://www.mdpi.com/article/10.3390/plants15172551/s1.
